# Dibromido(4,7-diazadecane-1,10-di­amine)­copper(II)

**DOI:** 10.1107/S160053681103251X

**Published:** 2011-08-17

**Authors:** Gervas E. Assey, Ray J. Butcher, Yilma Gultneh

**Affiliations:** a Department of Chemistry, Howard University, 525 College Street NW, Washington DC 20059, USA

## Abstract

In the title compound, [CuBr_2_(C_8_H_22_N_4_)], the Cu^II^ atom is six-coordinate forming a distorted octa­hedral complex and is bonded to two axial bromide anions and four equatorial nitro­gen donors. The equatorial Cu—N bond distances range from 2.005 (8) to 2.046 (8) Å while the axial Cu—Br distances are 2.8616 (17) and 2.9402 (17) Å, thus the six-coordinate Cu complex shows the usual Jahn–Teller distortion. All amine hydrogen atoms participate in either inter- or intra­molecular hydrogen bonding to the Br anions.

## Related literature

For related structues, see: Lee *et al.* (1986 ▶). For other related literature, see: Jahn & Teller (1937 ▶).
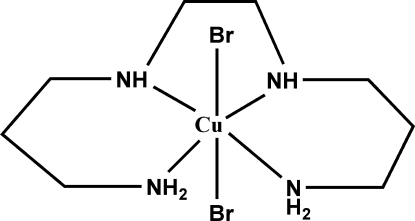


## Experimental

### 

#### Crystal data

[CuBr_2_(C_8_H_22_N_4_)]
*M* *_r_* = 397.66
Orthorhombic, 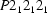

*a* = 6.9666 (4) Å
*b* = 8.4146 (6) Å
*c* = 24.0261 (15) Å
*V* = 1408.45 (15) Å^3^
*Z* = 4
Mo *K*α radiation
μ = 7.20 mm^−1^
*T* = 110 K
0.47 × 0.31 × 0.22 mm

#### Data collection

Oxford Diffraction Xcalibur diffractometer with a Ruby detector
Absorption correction: analytical (*CrysAlis PRO*; Oxford Diffraction, 2007 ▶) *T*_min_ = 0.157, *T*_max_ = 0.282
9561 measured reflections
2758 independent reflections
2262 reflections with *I* > 2σ(*I*)
*R*_int_ = 0.072

#### Refinement

*R*[*F* ^2^ > 2σ(*F* ^2^)] = 0.071
*wR*(*F* ^2^) = 0.183
*S* = 1.07
2758 reflections
136 parameters
24 restraints
H-atom parameters constrained
Δρ_max_ = 2.51 e Å^−3^
Δρ_min_ = −1.98 e Å^−3^

### 

Data collection: *CrysAlis PRO* (Oxford Diffraction, 2007 ▶); cell refinement: *CrysAlis PRO*; data reduction: *CrysAlis RED* (Oxford Diffraction, 2007 ▶); program(s) used to solve structure: *SHELXS97* (Sheldrick, 2008 ▶); program(s) used to refine structure: *SHELXL97* (Sheldrick, 2008 ▶); molecular graphics: *SHELXTL* (Sheldrick, 2008 ▶); software used to prepare material for publication: *SHELXTL*.

## Supplementary Material

Crystal structure: contains datablock(s) I, global. DOI: 10.1107/S160053681103251X/pv2444sup1.cif

Structure factors: contains datablock(s) I. DOI: 10.1107/S160053681103251X/pv2444Isup2.hkl

Additional supplementary materials:  crystallographic information; 3D view; checkCIF report

## Figures and Tables

**Table 1:** Hydrogen-bond geometry (Å, °)

*D*—H⋯*A*	*D*—H	H⋯*A*	*D*⋯*A*	*D*—H⋯*A*
N1—H1*C*⋯Br2^i^	0.92	2.66	3.466 (9)	147
N1—H1*D*⋯Br2	0.92	2.80	3.339 (8)	119
N2—H2*C*⋯Br1^ii^	0.93	2.66	3.407 (8)	138
N2—H2*C*⋯Br2	0.93	3.01	3.519 (7)	116
N3—H3*C*⋯Br1	0.93	2.90	3.409 (8)	116
N4—H4*C*⋯Br2^i^	0.92	2.60	3.515 (8)	171
N4—H4*D*⋯Br2^iii^	0.92	2.69	3.425 (8)	138
N4—H4*D*⋯Br1	0.92	2.94	3.433 (8)	115

Symmetry codes: (i) 
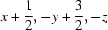
; (ii) 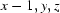; (iii) 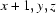.

**Fig. 1.**
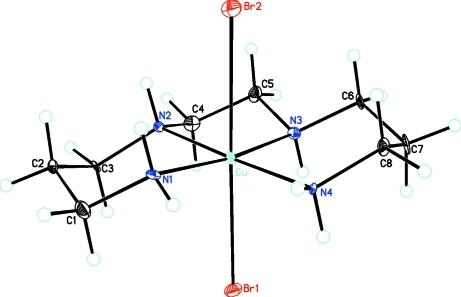
An ORTEP drawing of the title complex showing atom labeling. Thermal ellipsoids are drawn at the 30% probability level.

**Fig. 2.**
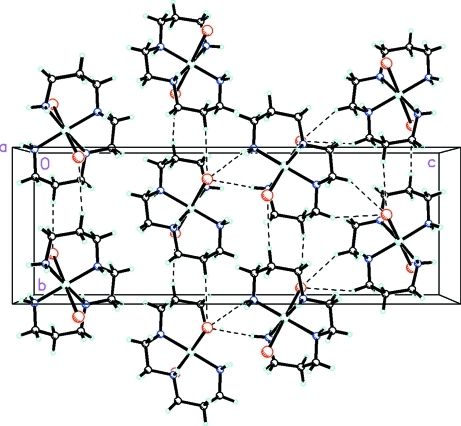
The molecular packing for the title compound viewed down the a axis. Hydrogen bonds are showed by dashed lines.

## supplementary crystallographic information

### Comment

In this study, the title compound was prepared and its structure determined by X-ray analysis. Owing to the Jahn-Teller distortion (Jahn & Teller, 1937), the Cu(II) center adopts an axially distorted octahedral CuN_4_Br_2_ conformation with the axial positions are occupied by the bromide anions. The equatorial positions are occupied by the N_4_ set of donor nitrogen atoms and the Cu1 lies in the N_4_ plane; maximum deviation of any atom from the mean-plane formed by CuN_4_ fragment being 0.042 (4) for N3. The structure of a related compound containing the same linear tetramine, has been reported (Lee *et al.* 1986) and its structural features compared with those of other linear Cu(II) aliphatic tetraamines of the type H_2_N(CH_2_)_l_NH-(CH_2_)_m_NH(CH_2_)_n_NH_2_ where l, m and n are 2 or 3. From this it can be seen that in the title complex, the equatorial Cu—N bond distances range from 2.005 (8) to 2.046 (8) Å and are in the normal range for such bonds. However, the axial Cu—Br distances are elongated at 2.8616 (17) and 2.9402 (17) Å, thus the 6-coordinate Cu complex shows the usual Jahn-Teller distortion. All amine H's participate in either inter or intramolecular hydrogen bonding to the Br anions.

### Experimental

The title compound was obtained as a byproduct of an attempt to prepare copper complexes of ethylenediamine *N*,*N*-bis(propylsalicylaldimine). A solution of N, *N*-bis(3-aminopropylethylene)diamine (5 g, 30.52 mmol) in methanol (20 ml) was added dropwise to a solution of salicylaldehyde (7.45 g, 61.04 mmol) in methanol (20 ml). The mixture was refluxed overnight while stirring with magnetic stirrer. Then the reaction mixture was evaporated under reduced pressure. An oily orange product was obtained which later solidified into a yellow compound, [2-(3-amino-propylamino)-ethyl]-propane-1,3-diamine-bis(salicyladimine), used as a ligand (H_2_*L*_4_) in the subsequent reaction. The synthesis of the title complex was achieved by the reaction of CuBr (1.5 g, 10.5 mmol) in methanol (20 ml) with of the ligand H_2_*L*_4_ (2 g, 5.2 mmol) dissolved in CH_2_Cl_2_ (25 ml). The ligand solution was added drop-wise to the solution of the metal salt and stirred at room temperature for 24 h. The mixture was then concentrated by evaporation under reduced pressure to afford a thick greenish liquid. Part of the complex was dissolved in dimethyl formamide (DMF), filtered and layered with diethyl ether for slow diffusion and X-ray quality crystals were obtained.

### Refinement

H atoms were placed in geometrically idealized positions and constrained to ride on their parent atoms with a C—H distance of 0.99 Å and N—H distances of 0.92 (primary amine) and 0.93 (secondary amine) with *U*_iso_(H) = 1.2*U*_eq_(C, N). Even though a face-indexed absorption correction was carried out, the thermal parameters for C3, C6, C7, and N4 atoms did not behave well and thus were restrained using ISOR command in SHELXL. The crystal was originally refined as a racemic twin with components 0.87 (3):0.13 (3). However, as the absolute configuration was not established unambiguously, the data were merged. In addition, the highest peak (2.50 e^-^/Å^3^, 0.70 Å from Cu) and deepest hole (-1.98 e^-^/Å^3^, 0.54 Å from Br2) are indicative of the problems with both the racemic twinning and absorption effects.

### Crystal data

**Table:**

[CuBr_2_(C_8_H_22_N_4_)]	*F*(000) = 788
*M**_r_* = 397.66	*D*_x_ = 1.875 Mg m^−^^3^
Orthorhombic, *P*2_1_2_1_2_1_	Mo *K*α radiation, λ = 0.71073 Å
Hall symbol: P 2ac 2ab	Cell parameters from 4805 reflections
*a* = 6.9666 (4) Å	θ = 4.6–32.8°
*b* = 8.4146 (6) Å	µ = 7.20 mm^−^^1^
*c* = 24.0261 (15) Å	*T* = 110 K
*V* = 1408.45 (15) Å^3^	Prism, dark blue
*Z* = 4	0.47 × 0.31 × 0.22 mm

### Data collection

**Table:**

Goniometer Xcalibur, detector Ruby (Gemini Mo) diffractometer	2758 independent reflections
Radiation source: Enhance (Mo) X-ray Source	2262 reflections with *I* > 2σ(*I*)
graphite	*R*_int_ = 0.072
Detector resolution: 10.5081 pixels mm^-1^	θ_max_ = 32.8°, θ_min_ = 4.6°
ω scans	*h* = −10→9
Absorption correction: analytical (*CrysAlis PRO*; Oxford Diffraction, 2007)	*k* = −12→11
*T*_min_ = 0.157, *T*_max_ = 0.282	*l* = −36→35
9561 measured reflections	

### Refinement

**Table:**

Refinement on *F*^2^	Primary atom site location: structure-invariant direct methods
Least-squares matrix: full	Secondary atom site location: difference Fourier map
*R*[*F*^2^ > 2σ(*F*^2^)] = 0.071	Hydrogen site location: inferred from neighbouring sites
*wR*(*F*^2^) = 0.183	H-atom parameters constrained
*S* = 1.07	*w* = 1/[σ^2^(*F*_o_^2^) + (0.103*P*)^2^ + 8.8289*P*] where *P* = (*F*_o_^2^ + 2*F*_c_^2^)/3
2758 reflections	(Δ/σ)_max_ < 0.001
136 parameters	Δρ_max_ = 2.51 e Å^−^^3^
24 restraints	Δρ_min_ = −1.98 e Å^−^^3^

### Special details

**Table:**

Geometry. All s.u.'s (except the s.u. in the dihedral angle between two l.s. planes) are estimated using the full covariance matrix. The cell s.u.'s are taken into account individually in the estimation of s.u.'s in distances, angles and torsion angles; correlations between s.u.'s in cell parameters are only used when they are defined by crystal symmetry. An approximate (isotropic) treatment of cell s.u.'s is used for estimating s.u.'s involving l.s. planes.
Refinement. Refinement of *F*^2^ against ALL reflections. The weighted *R*-factor *wR* and goodness of fit *S* are based on *F*^2^, conventional *R*-factors *R* are based on *F*, with *F* set to zero for negative *F*^2^. The threshold expression of *F*^2^ > 2σ(*F*^2^) is used only for calculating *R*-factors(gt) *etc*. and is not relevant to the choice of reflections for refinement. *R*-factors based on *F*^2^ are statistically about twice as large as those based on *F*, and *R*- factors based on ALL data will be even larger.

### Fractional atomic coordinates and isotropic or equivalent isotropic displacement parameters (Å^2^)

**Table:**

	*x*	*y*	*z*	*U*_iso_*/*U*_eq_	
Cu	0.84387 (19)	0.88632 (13)	0.11140 (4)	0.0099 (2)	
Br1	1.15943 (15)	1.06828 (12)	0.15326 (4)	0.0187 (2)	
Br2	0.52369 (16)	0.69368 (14)	0.06892 (5)	0.0225 (3)	
N1	0.8009 (12)	1.0062 (10)	0.0394 (3)	0.0132 (16)	
H1C	0.9013	0.9823	0.0160	0.016*	
H1D	0.6914	0.9661	0.0233	0.016*	
N2	0.6456 (12)	1.0252 (8)	0.1509 (3)	0.0106 (13)	
H2C	0.5256	0.9901	0.1392	0.013*	
N3	0.8538 (13)	0.7653 (9)	0.1852 (3)	0.0125 (14)	
H3C	0.9601	0.8035	0.2044	0.015*	
N4	1.0360 (12)	0.7415 (9)	0.0756 (3)	0.0110 (14)	
H4C	1.0215	0.7508	0.0376	0.013*	
H4D	1.1562	0.7796	0.0841	0.013*	
C1	0.7815 (17)	1.1841 (12)	0.0409 (4)	0.0178 (19)	
H1A	0.9031	1.2316	0.0542	0.021*	
H1B	0.7571	1.2240	0.0028	0.021*	
C2	0.6201 (14)	1.2343 (11)	0.0785 (4)	0.0143 (18)	
H2A	0.5018	1.1786	0.0666	0.017*	
H2B	0.5983	1.3496	0.0734	0.017*	
C3	0.6518 (15)	1.2020 (11)	0.1399 (4)	0.0123 (15)	
H3A	0.7780	1.2451	0.1514	0.015*	
H3B	0.5511	1.2559	0.1620	0.015*	
C4	0.6583 (17)	0.9909 (12)	0.2110 (4)	0.0160 (17)	
H4A	0.5407	1.0279	0.2302	0.019*	
H4B	0.7700	1.0467	0.2274	0.019*	
C5	0.6801 (16)	0.8160 (13)	0.2179 (4)	0.0179 (19)	
H5A	0.5644	0.7606	0.2039	0.021*	
H5B	0.6970	0.7891	0.2577	0.021*	
C6	0.8734 (14)	0.5888 (11)	0.1836 (4)	0.0138 (18)	
H6A	0.7584	0.5421	0.1658	0.017*	
H6B	0.8813	0.5473	0.2221	0.017*	
C7	1.0503 (15)	0.5394 (11)	0.1517 (4)	0.0158 (18)	
H7A	1.0739	0.4248	0.1581	0.019*	
H7B	1.1623	0.5986	0.1663	0.019*	
C8	1.0339 (15)	0.5691 (11)	0.0892 (4)	0.0142 (17)	
H8A	1.1419	0.5161	0.0700	0.017*	
H8B	0.9130	0.5216	0.0754	0.017*	

### Atomic displacement parameters (Å^2^)

**Table:**

	*U*^11^	*U*^22^	*U*^33^	*U*^12^	*U*^13^	*U*^23^
Cu	0.0119 (5)	0.0064 (4)	0.0113 (4)	0.0022 (4)	0.0026 (4)	0.0007 (4)
Br1	0.0107 (4)	0.0185 (5)	0.0270 (5)	−0.0016 (4)	−0.0007 (4)	−0.0062 (4)
Br2	0.0169 (5)	0.0213 (5)	0.0293 (5)	−0.0013 (4)	−0.0023 (4)	−0.0026 (4)
N1	0.011 (4)	0.011 (4)	0.017 (4)	−0.004 (3)	−0.001 (3)	0.002 (3)
N2	0.010 (3)	0.005 (3)	0.017 (3)	0.001 (3)	−0.001 (3)	−0.001 (3)
N3	0.011 (3)	0.012 (3)	0.015 (3)	−0.001 (3)	0.003 (3)	0.002 (3)
N4	0.008 (3)	0.009 (3)	0.016 (3)	0.002 (2)	0.002 (2)	0.000 (2)
C1	0.023 (5)	0.012 (4)	0.018 (4)	−0.002 (4)	0.003 (4)	0.005 (4)
C2	0.012 (4)	0.005 (3)	0.026 (5)	0.004 (3)	0.001 (3)	0.002 (3)
C3	0.012 (3)	0.006 (3)	0.019 (3)	0.001 (3)	0.001 (3)	0.002 (3)
C4	0.017 (4)	0.021 (4)	0.010 (3)	0.002 (4)	0.004 (4)	−0.002 (3)
C5	0.019 (5)	0.020 (4)	0.015 (4)	0.006 (4)	0.006 (4)	0.003 (4)
C6	0.016 (4)	0.009 (3)	0.017 (3)	0.003 (3)	0.002 (3)	0.004 (3)
C7	0.020 (4)	0.009 (3)	0.018 (3)	0.005 (3)	0.001 (3)	0.003 (3)
C8	0.016 (4)	0.009 (4)	0.017 (4)	0.004 (4)	0.004 (3)	−0.003 (3)

### Geometric parameters (Å, °)

**Table:**

Cu—N4	2.005 (8)	C1—H1B	0.9900
Cu—N1	2.025 (8)	C2—C3	1.517 (13)
Cu—N2	2.043 (8)	C2—H2A	0.9900
Cu—N3	2.046 (8)	C2—H2B	0.9900
Cu—Br1	2.8616 (17)	C3—H3A	0.9900
Cu—Br2	2.9402 (17)	C3—H3B	0.9900
N1—C1	1.503 (13)	C4—C5	1.489 (15)
N1—H1C	0.9200	C4—H4A	0.9900
N1—H1D	0.9200	C4—H4B	0.9900
N2—C4	1.476 (12)	C5—H5A	0.9900
N2—C3	1.512 (11)	C5—H5B	0.9900
N2—H2C	0.9300	C6—C7	1.510 (14)
N3—C6	1.492 (11)	C6—H6A	0.9900
N3—C5	1.504 (13)	C6—H6B	0.9900
N3—H3C	0.9300	C7—C8	1.526 (14)
N4—C8	1.487 (12)	C7—H7A	0.9900
N4—H4C	0.9200	C7—H7B	0.9900
N4—H4D	0.9200	C8—H8A	0.9900
C1—C2	1.503 (14)	C8—H8B	0.9900
C1—H1A	0.9900		
			
N4—Cu—N1	92.0 (3)	H1A—C1—H1B	108.0
N4—Cu—N2	177.1 (3)	C1—C2—C3	115.2 (8)
N1—Cu—N2	90.7 (3)	C1—C2—H2A	108.5
N4—Cu—N3	92.7 (3)	C3—C2—H2A	108.5
N1—Cu—N3	173.4 (4)	C1—C2—H2B	108.5
N2—Cu—N3	84.6 (3)	C3—C2—H2B	108.5
N4—Cu—Br1	87.9 (2)	H2A—C2—H2B	107.5
N1—Cu—Br1	98.5 (2)	N2—C3—C2	110.0 (7)
N2—Cu—Br1	92.9 (2)	N2—C3—H3A	109.7
N3—Cu—Br1	86.3 (3)	C2—C3—H3A	109.7
N4—Cu—Br2	91.3 (2)	N2—C3—H3B	109.7
N1—Cu—Br2	82.3 (2)	C2—C3—H3B	109.7
N2—Cu—Br2	87.9 (2)	H3A—C3—H3B	108.2
N3—Cu—Br2	93.0 (3)	N2—C4—C5	107.9 (8)
Br1—Cu—Br2	178.89 (6)	N2—C4—H4A	110.1
C1—N1—Cu	119.3 (7)	C5—C4—H4A	110.1
C1—N1—H1C	107.5	N2—C4—H4B	110.1
Cu—N1—H1C	107.5	C5—C4—H4B	110.1
C1—N1—H1D	107.5	H4A—C4—H4B	108.4
Cu—N1—H1D	107.5	C4—C5—N3	107.7 (9)
H1C—N1—H1D	107.0	C4—C5—H5A	110.2
C4—N2—C3	111.2 (7)	N3—C5—H5A	110.2
C4—N2—Cu	107.6 (6)	C4—C5—H5B	110.2
C3—N2—Cu	117.6 (6)	N3—C5—H5B	110.2
C4—N2—H2C	106.6	H5A—C5—H5B	108.5
C3—N2—H2C	106.6	N3—C6—C7	111.2 (8)
Cu—N2—H2C	106.6	N3—C6—H6A	109.4
C6—N3—C5	111.7 (8)	C7—C6—H6A	109.4
C6—N3—Cu	118.5 (6)	N3—C6—H6B	109.4
C5—N3—Cu	106.5 (6)	C7—C6—H6B	109.4
C6—N3—H3C	106.5	H6A—C6—H6B	108.0
C5—N3—H3C	106.5	C6—C7—C8	113.1 (8)
Cu—N3—H3C	106.5	C6—C7—H7A	109.0
C8—N4—Cu	119.4 (6)	C8—C7—H7A	109.0
C8—N4—H4C	107.5	C6—C7—H7B	109.0
Cu—N4—H4C	107.5	C8—C7—H7B	109.0
C8—N4—H4D	107.5	H7A—C7—H7B	107.8
Cu—N4—H4D	107.5	N4—C8—C7	112.1 (8)
H4C—N4—H4D	107.0	N4—C8—H8A	109.2
C2—C1—N1	111.2 (8)	C7—C8—H8A	109.2
C2—C1—H1A	109.4	N4—C8—H8B	109.2
N1—C1—H1A	109.4	C7—C8—H8B	109.2
C2—C1—H1B	109.4	H8A—C8—H8B	107.9
N1—C1—H1B	109.4		
			
N4—Cu—N1—C1	−139.1 (8)	N1—Cu—N4—C8	−138.9 (7)
N2—Cu—N1—C1	42.1 (8)	N3—Cu—N4—C8	36.5 (7)
Br1—Cu—N1—C1	−50.9 (8)	Br1—Cu—N4—C8	122.7 (7)
Br2—Cu—N1—C1	129.9 (8)	Br2—Cu—N4—C8	−56.6 (7)
N1—Cu—N2—C4	−170.1 (6)	Cu—N1—C1—C2	−57.3 (11)
N3—Cu—N2—C4	14.4 (6)	N1—C1—C2—C3	67.5 (11)
Br1—Cu—N2—C4	−71.6 (6)	C4—N2—C3—C2	−174.8 (8)
Br2—Cu—N2—C4	107.6 (6)	Cu—N2—C3—C2	60.6 (10)
N1—Cu—N2—C3	−43.7 (7)	C1—C2—C3—N2	−69.7 (11)
N3—Cu—N2—C3	140.8 (7)	C3—N2—C4—C5	−171.9 (9)
Br1—Cu—N2—C3	54.8 (6)	Cu—N2—C4—C5	−41.8 (10)
Br2—Cu—N2—C3	−126.0 (6)	N2—C4—C5—N3	56.4 (11)
N4—Cu—N3—C6	−37.2 (8)	C6—N3—C5—C4	−172.8 (8)
N2—Cu—N3—C6	141.9 (8)	Cu—N3—C5—C4	−42.0 (9)
Br1—Cu—N3—C6	−124.9 (7)	C5—N3—C6—C7	−178.8 (8)
Br2—Cu—N3—C6	54.3 (7)	Cu—N3—C6—C7	56.9 (10)
N4—Cu—N3—C5	−164.0 (7)	N3—C6—C7—C8	−70.4 (11)
N2—Cu—N3—C5	15.1 (6)	Cu—N4—C8—C7	−55.5 (10)
Br1—Cu—N3—C5	108.3 (6)	C6—C7—C8—N4	69.7 (11)
Br2—Cu—N3—C5	−72.5 (6)		

### Hydrogen-bond geometry (Å, °)

**Table:**

*D*—H···*A*	*D*—H	H···*A*	*D*···*A*	*D*—H···*A*
N1—H1C···Br2^i^	0.92	2.66	3.466 (9)	147.
N1—H1D···Br2	0.92	2.80	3.339 (8)	119.
N2—H2C···Br1^ii^	0.93	2.66	3.407 (8)	138.
N2—H2C···Br2	0.93	3.01	3.519 (7)	116.
N3—H3C···Br1	0.93	2.90	3.409 (8)	116.
N4—H4C···Br2^i^	0.92	2.60	3.515 (8)	171.
N4—H4D···Br2^iii^	0.92	2.69	3.425 (8)	138.
N4—H4D···Br1	0.92	2.94	3.433 (8)	115.

Symmetry codes: (i) *x*+1/2, −*y*+3/2, −*z*; (ii) *x*−1, *y*, *z*; (iii) *x*+1, *y*, *z*.
